# The Role of RSV Infection in Asthma Initiation and Progression: Findings in a Mouse Model

**DOI:** 10.1155/2011/748038

**Published:** 2011-07-02

**Authors:** Junyan Han, Katsuyuki Takeda, Erwin W. Gelfand

**Affiliations:** Division of Cell Biology, Department of Pediatrics, National Jewish Health, Denver, CO 80206, USA

## Abstract

Respiratory syncytial virus (RSV) is a common cause of severe lower respiratory tract diseases (bronchiolitis and pneumonia) during infancy and early childhood. There is increasing evidence which indicates that severe pulmonary disease caused by RSV infection in infancy is associated with recurrent wheezing and development of asthma later in childhood. However, the underlying mechanisms linking RSV infection to persistent airway hyperresponsiveness and dysfunction are not fully defined. To study these processes in ways which are not available in humans, animal models have been established and have provided valuable insight into the pathophysiology of RSV-induced disease. In this paper, we discuss experimental models of RSV infection in mice and highlight a new investigative approach in which mice are initially infected as neonates and then reinfected later in life. The findings shed light on the mechanisms underlying the association between early severe RSV infection and development of asthma later in childhood.

## 1. Introduction

Respiratory syncytial virus (RSV) is the leading cause of viral lower respiratory tract infections (bronchiolitis and pneumonia) during infancy and early childhood worldwide. About 65% of children are infected with RSV within the first year of life, and almost all children are infected at least once by 2 years of age. Notably, repeated infection is common at all ages; however, therapeutic options are limited and somewhat ineffective. 

Infants hospitalized with RSV-associated bronchiolitis are at increased risk of recurrent wheezing and asthma later in childhood [1]. However, in nonhospitalized patients, the relationship is less clear [2], and indeed other viruses, such as rhinovirus, have also shown predisposing attributes [3]. The underlying mechanisms responsible for the interactions between RSV infection and the development of asthma are controversial. In general, animal disease models provide opportunities to address issues such as underlying mechanisms or potential therapeutic interventions that are impossible to study in humans, especially when the target population is the very young. Due to the variable consequences of RSV-induced bronchiolitis, especially in predisposed atopic compared to nonatopic infants, animal models have been developed in an attempt to mimic the clinical conditions. Here, we briefly discuss mouse models of RSV infection and a new investigative model with neonatally infected mice that may elucidate the mechanisms underlying the association between early severe RSV infection and development of asthma later in childhood.

## 2. Animal Models of RSV Disease

Many species have been used to develop animal models of human RSV disease including chimpanzees, calves, cotton rats, guinea pigs, ferrets, hamsters, and mice [4]. Each of these models has both inherent advantages and disadvantages. No single animal model duplicates all forms of human RSV-related disease. When choosing a suitable animal model, two factors must be considered. First, the animal species should be susceptible to the virus, allowing infection and replication. Second, the animal model should mirror at least some aspects of human disease. The animal models have contributed to understanding the pathogenesis of RSV infection to some degree and have been most useful in testing drugs that inhibit virus replication. Mouse models are the most widely used since (a) from an immunological standpoint, mice are the best characterized animals and a large pool of immunological reagents are available, (b) their body size is small and the breeding period is short so experiments can be performed in a cost- and time-effective manner, (c) a wide array of gene-manipulated mice (transgenic or knockout mouse strains) have been generated which allow direct assessment of the roles of single genes or related proteins in development of disease, and (d) mouse models have been widely used to investigate asthma, an advantage to study the interaction between RSV infection and allergic airway responses. 

RSV is a negative-sense, single-stranded RNA virus of the paramyxovirus subfamily pneumovirinae. The single greatest disadvantage in using mouse models for studies of the human RSV pathogen is that pneumoviruses are highly restricted to their natural hosts. Human RSV is not a natural rodent pathogen; therefore, a large inoculum of virus is required to achieve even a modest degree of virus replication. In the most permissive mouse strains, such as BALB/c, challenge using an inoculum (1–10 million plaque-forming units (pfu)) of human RSV wild-type (WT) strain A2 or the WT long strain is required to achieve productive infection. A high dose (10^7^–10^8^ pfu) may cause severe alveolitis with pneumonia, whereas a lower dose (10^5^–10^6^ pfu) mainly causes bronchiolitis with no alveolitis or pneumonia. For these reasons, some groups have used pneumonia virus of mice (PVM), the mouse equivalent strain of human RSV. Because PVM is a natural rodent pathogen, far fewer virions are required to induce infection. PVM replicates to high titers in the mouse lung but can cause severe disease with significant mortality in immunocompetent hosts, which is rarely seen in humans. As little as 2 pfu elicits a respiratory tract infection, ~30 pfu provokes a productive infection with severe bronchiolitis, and 300 pfu results in rapid death [5]. However, antiviral therapies specific to human viruses (e.g., neutralizing antibodies) cannot be tested in these models using nonhuman viruses.

Most of the studies with mouse models were initiated by intranasal inoculation of human RSV. Viral replication, airway inflammation, innate and adaptive immunity, and airway responsiveness are measured at different time points after RSV infection. Virus replication in the lung usually peaks 3-4 days after infection, and clearance is generally achieved by 2-3 weeks after inoculation. However, evidence for RSV RNA which persisted in lung homogenates for 100 days or more has recently been demonstrated [6, 7]. The significance of such virus persistence is not clear, and it is not known whether the persisting virus can cause any form of chronic airway disease. In general, RSV infection can induce secretion of cytokines such as IFN-*γ*, IL-4, IL-5, IL-10, IL-12, IL-13, and chemokines such as CCL2, CCL3, CCL5, CXCL10, and KC [8]. RSV infection also induces the generation of lipid inflammatory mediators, such as cysteinyl leukotrienes [9, 10]. Many immune and inflammatory cells are involved in acute RSV infection, including neutrophils, dendritic cells (DCs), macrophages, natural killer (NK) cells, natural killer T (NKT) cells, T cells, and eosinophils. It has been recognized that the host immune responses to RSV are responsible for a substantial portion of RSV-induced pathophysiological changes [11–13]. In the context of this discussion, RSV infection induces airway hyperresponsiveness (AHR) to inhaled methacholine (MCh) [14, 15]. Schwarze et al. showed that acute RSV infection in mice results in AHR and neutrophilic and eosinophilic inflammation [14]. This response is associated with the predominant production of Th1-type cytokines from peribronchial lymph node cells in vitro. The development of AHR to inhaled MCh peaked on day 7 after RSV infection [16], and 14–21 days after RSV infection, AHR resolved to baseline levels. Epithelial cell damage, inflammation, and neural pathways have been shown to be involved in RSV-induced AHR in mice [17, 18].

The extent and duration of lung histopathology may vary depending on the particular model selected. A number of factors must be taken into consideration when interpreting data from these different models: (a) the dose of virus inoculum, (b) the strain and purity of the virus: the virus preparation should be purified and inoculated free of culture-derived factors (cytokines, chemokines, etc.) that could potentially modify the host's response to the virus. (c) The route of inoculation: in most studies, RSV is given intranasally, but in some animal models RSV is given intratracheally. Intratracheal inoculation may require a lower dose of RSV to achieve similar lung histopathology. (d) The strain of mouse used: BALB/c mice are the most commonly used strain because they appear to be the most permissive for human RSV. However, BALB/c mice tend to be more proficient in producing Th2 responses than other strains such as C57BL/6 mice [19]. (e) The last factor is the age of mice at the point of viral inoculation. 

## 3. Mouse Models of Asthma

Asthma is a complex syndrome of unknown etiology. Mouse models are the most commonly used animal model to study the pathogenesis of asthma. Experimental asthma is usually induced by initial systemic sensitization to allergen together with alum as an adjuvant followed by airway challenge with aerosolized allergen for 1–9 days in acute models and for 5–9 weeks in chronic models. In these models, different strains of mice (with different susceptibilities) can be used. In addition, the mode of sensitization (with or without adjuvant), the route of sensitization (systemic or local airway sensitization), and the frequency and duration of airway allergen challenge can be varied, leading to identification of various pathways and mechanisms that perhaps reflect the heterogeneity of human asthma [20, 21]. 

To investigate the interaction of RSV infection and asthma, these experimental asthma models are combined with RSV infection, either prior to or following allergen challenge, uncovering mechanisms whereby viral infections may contribute to the pathophysiology of asthma.

## 4. Effects of Prior Airway Allergen Exposure on RSV-Induced Airway Responses

Following RSV infection, most individuals develop mild symptoms, which are usually restricted to the upper airways [22]. This is in contrast to subjects with underlying diseases such as asthma, where the virus may take advantage of a deficient antiviral response and spread to the lower airways where they mediate tissue damage and inflammation. This can result in exaggerated constrictive airway responses to both allergen and nonspecific stimuli [23]. Indeed, a viral infection is the most common cause of an asthma exacerbation [24]. 

In earlier studies, when BALB/c mice were previously exposed to OVA via the airways followed by RSV infection, the degree of AHR was significantly increased and was associated with an increased proportion of Th2 (IL-4, IL-5) cytokine-producing T lymphocytes [25]. This response was also associated with the increased accumulation of eosinophils, neutrophils, and CD8+ T cells in the lungs. These results suggested that airway allergen exposure prior to RSV infection may predispose sensitized hosts to a greater degree of RSV-induced AHR and airway inflammation. Similarly, when using a different trigger, ultrafine carbon black (CB) particles, Lambert et al. demonstrated a synergistic effect of ultrafine CB particles with RSV infection on the development of airway inflammation and AHR in BALB/c mice [26]. These data indicate that prior exposure to allergen in a sensitized host can lead to enhanced Th2-dominant immune responses against RSV. Among the Th2 cytokines released following RSV infection in “allergic” animals, IL-4, IL-5, and IL-13 are involved in development of airway eosinophilia and AHR [25, 27, 28]. 

## 5. Effects of Initial RSV Infection on Subsequent Airway Allergic Responses

It appears that RSV infection, particularly in children under 1 year old with severe bronchiolitis requiring hospitalization, has a strong linkage to development of allergic asthma [29–31]. An ongoing debate that remains unresolved is whether RSV can induce asthma and whether the response is different in atopic compared to nonatopic infants. Certainly, the presence of IgE antibodies to RSV has been linked to more severe disease [32]. The development of clinical strategies is in many ways tied to our thinking. For example, the prevention of RSV lower respiratory tract infection, with the use of anti-RSV monoclonal antibody therapy, has shown some benefit in “preventing” asthma in some but not all studies [33]. If RSV is truly an initiator of asthma, then RSV preventive interventions could reduce or, at a minimum, delay the onset of asthma. Such interventions might be most effective among those genetically predisposed to more severe RSV infection and asthma. A recent study demonstrated that RSV prophylaxis in nonatopic children decreases the relative risk of recurrent wheezing by up to 80% but has little to no effect in infants with an atopic family history [34]. Although remaining to be confirmed, the findings suggest that RSV may predispose to recurrent wheezing in an atopy-independent manner. Two recent clinical studies addressing causation between infant RSV infection and asthma came to different conclusions. Wu and colleagues found that 4-month-old infants at the winter virus peak, which means that they likely were exposed to an entire RSV season at an age when they were most susceptible to develop severe RSV-associated disease, had an increased risk of bronchiolitis in infancy and then of asthma during childhood [35]. By contrast, Thomsen and colleagues, using a large dataset of 8,280 twin pairs in Denmark, found that the association between RSV and asthma was essentially due to shared genetic predisposition [36]. However, both studies suffered from inadequate phenotypic definition of RSV infection and of asthma and failed to shed further light on the continuing debate. 

Despite the many limitations of animal studies, controlled experiments can be carried out to directly determine the interplay between RSV and allergic sensitization and the development of an asthma-like phenotype. When RSV is administered after allergen exposure, this combination results in the development of a greater degree of AHR as described above [25, 26]. Barends et al. demonstrated that RSV infection during the provocation phase to allergen enhanced pulmonary expression of Th2 cytokines, lung pathologic lesions, and AHR but not Th1 responses [37]. The same group further demonstrated that the timing of RSV infection was critical for such RSV-enhanced allergic responses. When the immune system was Th2-primed by allergen (i.e., OVA), inoculation with RSV enhanced the allergic responses [38]. This is consistent with substantial clinical and epidemiologic evidence that RSV infection triggers exacerbations of asthma. Strong links between viral infection and acute worsening of asthma have been shown in approximately 80% of cases in children [39] and 70% in adults [40]. 

However, when RSV infection precedes airway allergen exposure, the experimental results are inconsistent. In an approach where OVA aerosolization began 10 days after RSV infection in BALB/c mice, Schwarze et al. demonstrated that mice sensitized to OVA via the airways after RSV infection developed increased AHR, and pulmonary eosinophilic and neutrophilic inflammation, associated with the predominant production of Th2-type cytokines [14]. Furthermore, treatment with anti-IL-5 antibody abolished AHR and eosinophilic but not neutrophilic inflammation in both RSV-infected and RSV-infected followed by OVA exposure groups. Freihorst et al. [41] showed significantly higher OVA-specific IgG in RSV-infected, allergen challenged BALB/c mice than the uninfected, OVA-challenged controls. Similar results have been reported in guinea pigs and calves [42, 43]. In contrast, Peebles et al. showed that RSV infection prior to allergic sensitization protected against the development of allergen-induced AHR and allergic airway inflammation [44]. Liu and Kimura observed similar results when using Japanese cedar pollen (JCP) as the allergen [45]. These discrepancies may reflect the complexity of RSV infection, differences among virus strains, and the approach to infection, all which can lead to differences in the extent of Th1 and Th2 responses. The genetic background of the mice, age at infection or allergen exposure, prior exposure to RSV, or allergen or microbial infections may all contribute to the differences in Th1/Th2 balance. Together, the data suggest that the airway response to allergen may depend on the host's immune setting induced by the prior RSV infection.

To mimic the human clinical situation more closely, some groups have employed an approach where BALB/c mice were neonatally infected with PVM followed by OVA sensitization and challenge, but after the airways were fully recovered from virus infection [46]. In this model, they found that early-life virus infection or allergen sensitization and challenge were independent variables capable of contributing to development of childhood asthma and that these interactions between early-life viral infection and allergen sensitization/challenge were essential for the full development of the characteristic features of childhood asthma, including eosinophilic inflammation and a Th2-biased immune response. This may explain how PVM, on its own or in conjunction with allergic sensitization, triggers an asthma-like phenotype. 

In addition to OVA, house dust mite (HDM), a common allergen in asthma, has been used to induce experimental models of asthma in mice. Studies examining the effects of RSV infection in HDM-exposed animals are less common. In one, RSV infection 3 days before HDM sensitization augmented Der f-specific antibody production and increased the number of blood eosinophils [47]. As in OVA models, RSV infection exacerbated pulmonary allergic responses to subsequent HDM exposure [48]. Recurrent RSV infections after HDM sensitization augmented synthesis of Th2 cytokines, total serum IgE, and MIP-1*α*, which resulted in persistent airway inflammation and airway hyperresponsiveness [49, 50]. 

## 6. Characterization of the Consequences of RSV Reinfection

Compared with other respiratory viruses, RSV infections appear more frequent and often more severe early in life. RSV has been implicated in mortality in the elderly [51]. The peak for hospitalization with RSV is at 2 months of age [22]. Infants at this young age have narrower airways and are more susceptible to obstruction, a prominent feature of RSV disease. Immune responses in general are lower in magnitude and potentially less effective in young infants than in older children or adults. In addition, there is a Th2 bias in immune response during the neonatal period [52, 53]. Compared to controls, a higher frequency of IL-4 producing T-cells responding to RSV and cat antigens were found in children who were hospitalized with RSV bronchiolitis in infancy [54]. RSV infection during the first 3 months of life induces a Th2-biased response compared to infection in older infants [55]. As infancy is a time of rapid lung maturation, severe airway inflammation may alter lung structure permanently and result in airway remodeling. Taken together, all of these features point to a period of increased susceptibility in early infancy. Interestingly, this also holds true for other viruses, such as human parainfluenza virus (HPIV) or influenza virus and thus may be more related to host age rather than any particular feature of the virus [55]. However, since RSV infects infants very early in life, the overall impact appears greater for RSV. This age effect appears to be true for allergic sensitization as well. In this regard, allergen sensitization at early age establishes long-lasting memory T cell responses which can be activated after airway allergen challenge causing AHR months after initial sensitization in mice [56]. 

**Figure 1:**
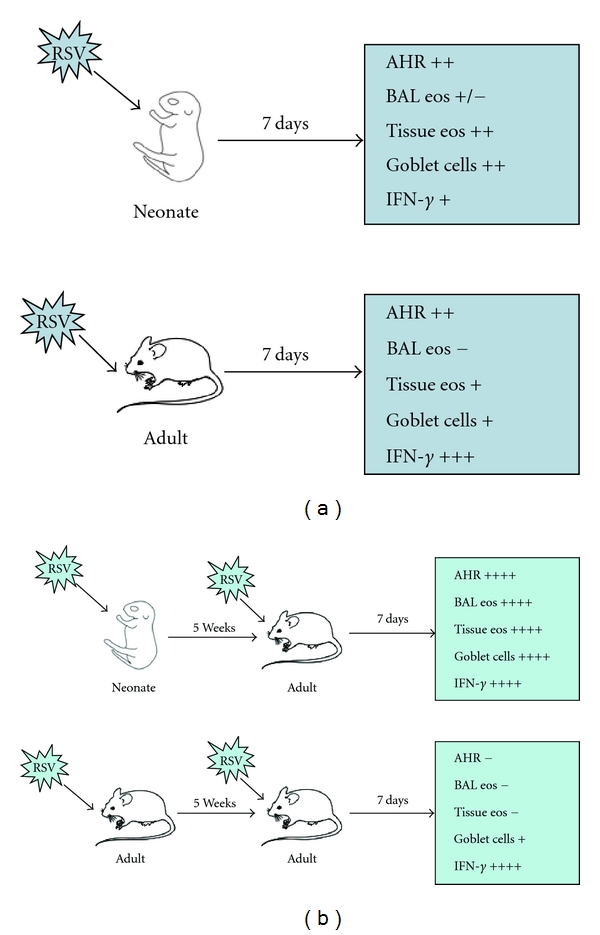
Age-related differences in airway responses. (a) Primary infection, (b) secondary infection.

An important aspect of RSV infection is that reinfection is common in children and is associated with wheezing and asthma after an episode of bronchiolitis [22]. Infection in a Th2-biased environment has the potential to affect the overall quality and nature of both primary and recall responses. Once infants become sensitized to RSV and a long-lasting type 2 immune response is established in the airways, they may go on to develop recurrent airway dysfunction (wheezing and perhaps asthma) on re-exposure to RSV. Reinfection with RSV has been examined in mice. Culley et al. found that primary infection of neonatal mice (during the first week of life) with RSV was associated with reduced and delayed IFN-*γ* responses [57]. Upon reinfection, these mice developed more severe weight loss with a Th2-biased lymphocyte response and airway eosinophilia compared with mice initially infected with RSV at an older age. We adopted a similar approach to examine the consequences of reinfection with RSV and to determine how the responses differ if initial infection was in neonates or weanling animals, in particular examining the consequences on airway responsiveness [16]. We showed that both age groups can develop significant AHR after primary RSV infection either at 1 or 3 weeks of age. When re-infected with RSV 5 weeks later, the two age groups developed very different airway responses. Initial RSV infection at 3 weeks of age elicited a protective airway response upon reinfection characterized by an increased airway inflammatory response consisting primarily of lymphocytes, but without development of AHR, eosinophilia, or mucus hyperproduction. In contrast, initial neonatal infection failed to protect the airways on reinfection and resulted in enhanced AHR, increased IL-13 production associated with mucus hyperproduction and airway eosinophilia (Figure 1) [16]. Moreover, IL-13 was shown to be critical to the development of the asthma-like phenotype after reinfection of mice initially infected as newborns. 

The underlying mechanisms responsible for these significant age differences are not entirely defined. In infants, an association between severity of RSV bronchiolitis and deficient IFN-*γ* production has been demonstrated [58–61]. Infants hospitalized for severe lower respiratory tract illness due to RSV infection had lower IFN-*γ* production from blood mononuclear cells compared to those with milder illnesses [58, 59]. Moreover, the levels of IFN-*γ* measured in nasopharyngeal aspirates were lower in infants hospitalized for severe RSV bronchiolitis compared to those exhibiting milder disease. These clinical studies suggested that IFN-*γ* plays an important role in determining the outcome of RSV-mediated disease. Similarly, in animal models, we and others found that neonates demonstrated lower IFN-*γ* responses to initial RSV infection compared with weanling or adult mice [16, 57]. The protective role of IFN-*γ* was confirmed in the RSV reinfection model using IFN-*γ*-deficient mice [62]. Here, when initial infection and reinfection were carried out in adult mice, IFN-*γ*-deficient mice developed significantly greater AHR, airway eosinophilia, and mucus hyperproduction following reinfection compared to the WT mice, regardless of the absence of significant differences in the response to initial infection. Provision of IFN-*γ* during primary neonatal infection prevented the development of enhanced AHR and lung histopathology upon reinfection with RSV. These results indicated a critical role for IFN-*γ* during the initial infection stage that dictated the subsequent outcomes of reinfection with RSV. This may also be true in humans so that provision of IFN-*γ* in infancy may interfere with the development of altered airway responses on reinfection at a later age. 

Specific IgE antibodies against viral pathogens have been identified in clinical studies following different viral infections, including RSV [32]. In most cases, development of virus-specific IgE has been associated with a more severe disease outcome [63, 64]. In the mouse model, primary RSV infection can also lead to the production of RSV-specific IgE, which may contribute to the development of exaggerated airway responses upon reinfection. Thus, there is a certain parallelism between RSV-mediated wheezing and allergen-triggered asthma. In the mouse reinfection model, we demonstrated that RSV-specific IgE enhances the development of Th2-biased airway responsiveness on reinfection of mice initially infected as neonates [65]. How RSV infection leads to an increase in RSV-specific IgE production, a hallmark of Th2 responses, and enhancement of disease is not clear. Recent studies using mouse Sendai virus (SeV) infection sheds new light on the potential mechanisms [66]. These studies showed that infection with mouse SeV led to increased expression of the high-affinity IgE receptor (Fc*ε*RI) on lung DCs, and this was type I interferon receptor dependent. Crosslinking of Fc*ε*RI on DCs resulted in CCL28 production, subsequent recruitment of IL-13-producing CD4+ T cells and development of mucus metaplasia. Thus, the development of virus-specific IgE may play a critical role in reinfection-induced allergic airway inflammation and AHR.

## 7. Conclusions

There is now sufficient evidence supporting the notion that severe infantile RSV infection is associated with recurrent wheezing and asthma later in childhood. However, the underlying mechanisms are not fully defined nor are the findings always consistent, perhaps suggesting genetic and environmental risk factors playing a role in dictating final outcomes. In early life, the tendency towards Th2-biased responses is likely an important variable, especially in hosts that are atopic or predisposed to becoming atopic. RSV infection in neonates may bias both the systemic immune response and the response in the lung. This in turn could lead to the development of asthma-like symptoms when re-exposed to RSV. Further, this Th2-biased setting seen with RSV reinfection and the development of IgE-specific antibodies may induce some cross-sensitization or lower the threshold to allergen-induced responses. This combination may manifest as increased asthma susceptibility. Studies in the mouse, although not complete surrogates of human disease, have provided valuable information in understanding the potential links between RSV infection and asthma.
